# Acetaminophen changes paracellular transport activity through regulation of the tight junction protein in an intestinal barrier model

**DOI:** 10.1186/2050-6511-13-S1-A12

**Published:** 2012-09-17

**Authors:** Mohammad R Lornejad-Schäfer, Christine Schäfer, Klaus R Schröder

**Affiliations:** 1 BioMed-zet Life Science GmbH, zet-LSL, 4020 Linz, Austria; 2 zet-Centre for Alternative and Complementary Methods to Animal Testing, 4020 Linz, Austria

## Background

Drugs and diet interact with each other in mutually influencing their bioavailability. In our previous work we have shown that *N*-acetyl-*p*-aminophenol (APAP, acetaminophen) reduces the paracellular transport activities of itself and co-administered substances. The aim of this study was to find out how APAP reduces the bioavailability of small molecules which pass paracellularly, and which tight junction proteins are involved in the regulation of paracellular transport using a Caco-2 barrier model.

## Methods

The Caco-2 *in vitro* model is widely used by the pharmaceutical industry to predict the absorption of orally administered drugs. To construct a Caco-2 barrier model, the Caco-2 cell line, a continuous line of heterogeneous human epithelial colorectal adenocarcinoma cells, was seeded onto inserts (Millipore, 0.4 µm pore size) for 21 days. After differentiation, the cells were treated topically with 10 mM APAP for 24 h. The cell transepithelial electrical resistance (TER) and capacitance (Ccl) were determined by two different impedance-measuring systems. The membrane permeability was tested by differently sized molecules: Lucifer Yellow (520 Da), 3–5 kDa and 40 kDa Fluorescein thiocarbamoyl (FITC)-dextran. The tight junction proteins (ZO-1, occludin) were investigated using Western blot analysis and immunofluorescence staining.

## Results

APAP increased the TER value, *i.e.* membrane integrity, which correlated significantly with the decrease of permeability of the small molecules Lucifer Yellow (ca. 520 Da) and FITC-dextran (3–5 kDa) but not of large molecules (40 kDa FITC-dextran). The tight junction protein ZO-1 and its tyrosine phosphorylation were up-regulated after 24 h treatment with APAP.

## Conclusions

Tight junction proteins play an important role in maintaining the intestinal barrier function. We assume that APAP affects the paracellular transport pathway through disassembly of the tight junction. We suppose that APAP leads to remodelling of the tight junction protein ZO-1 through changes in the level of protein expression and in tyrosine phosphorylation. APAP may reduce the bioavailability of co-administered substances through remodelling the tight junction by regulating ZO-1 and its tyrosine phosphorylation. As a result, the paracellular transport activity for small molecules is decreased as is net intestinal absorption.

